# Interpenetrated Polymer Network Systems (PEG/PNIPAAm) Using Gamma Irradiation: Biological Evaluation for Potential Biomedical Applications

**DOI:** 10.3390/ma17204998

**Published:** 2024-10-12

**Authors:** Angélica Cruz-Gómez, Guillermina Burillo, Daniel Perez-Calixto, Kenia Palomino, Héctor Magaña

**Affiliations:** 1 Instituto de Ciencias Nucleares, Universidad Nacional Autónoma de México, Ciudad Universitaria, Ciudad de México 04510, Mexico; gangelica.cg@ciencias.unam.mx (A.C.-G.); burillo@nucleares.unam.mx (G.B.); 2 Instituto Nacional de Medicina Genómica, Facultad de Ciencias, Universidad Nacional Autónoma de México, Ciudad Universitaria, Ciudad de México 04510, Mexico; daniel_perez@ciencias.unam.mx; 3 Facultad de Ciencias Químicas e Ingeniería, Universidad Autónoma de Baja California, Calzada Universidad 14418, Parque Industrial Internacional Tijuana, Tijuana 22390, Mexico; kenia.palomino@uabc.edu.mx

**Keywords:** interpenetrated polymer networks, medical devices, antifouling polymers, antimicrobial devices, biocompatibility materials

## Abstract

The potential antimicrobial and antibiofouling properties of previously synthesized PEG/NiPAAm interpenetrated polymer networks (IPNs) were investigated against three of the most common bacteria (*E. coli*, *S. aureus*, and *S. epidermidis*). The main goal was to evaluate the material’s biocompatibility and determine its potential use as an antifouling component in medical devices. This was intended to provide an alternative option that avoids drug usage as the primary treatment, thus contributing to the fight against antimicrobial resistance (AMR). Additionally, characterization and mechanical testing of the IPN were carried out to determine its resistance to manipulation processes in medical/surgical procedures. IPNs with different NiPAAm ratios exhibited excellent cytocompatibility with BALB/3T3 murine fibroblast cells, with cell viability values of between 90 and 98%. In addition, the results regarding the adsorption of albumin as a model protein showed a nearly constant adsorption percentage of almost zero. Furthermore, the bacterial inhibition tests yielded promising results, demonstrating effective pathogen growth inhibition after 48 h. These findings suggest the material’s suitability for use in biomedical applications.

## 1. Introduction

Some characteristics currently sought after in the development of biomaterials are high biocompatibility, chemical inertia, adequate mechanical properties (dimensional stability), and drug release control [1]. A biomaterial can be considered safe, stable, and practical if it satisfies these criteria. It can be submitted to the health regulatory agency of the relevant country for approval and commercialization [2]. Certain requirements must also be considered, such as those regarding its susceptibility to bacterial colonization and resistance [3]. Antimicrobial resistance (AMR) is a global issue. The World Health Organization (WHO) states that AMR ranks among the top 10 public health threats [4]. The main factors contributing to the evolution of drug-resistant pathogenic microbes include the indiscriminate use and abuse of antibiotics, insufficient education and skills among both patients and medical staff regarding wound treatment, and the prevalence and acquisition of intrahospital infections [5]. Given the rapid evolution of microbes, there are data suggest that a crisis is emerging, particularly considering that the development of new and effective drugs is a complex and time-consuming process. 

Consequently, the search for novel strategies to prevent and control diseases caused by these microorganisms has become a major priority. Nosocomial infectious agents can be easily transmitted due to patients’ exposure during their hospital stays and the use of essential medical materials and devices, such as surgical sutures, orthopedic implants, meshes, central venous catheters, urinary catheters, feeding tubes, endotracheal tubes, wound drains, wound dressings, etc. [6]. To mitigate unnecessary drug usage and AMR cases, researchers are pursuing the development of medical equipment with antibiofouling surfaces. This is based on the proposition that by preventing bacterial adhesion on devices, a reduction in health complications would be attainable, as this would limit the available space for pathogen colonization. Prominent pathogens, including *Escherichia coli, Staphylococcus aureus*, *Staphylococcus epidermidis*, *Enterococcus faecalis*, *Klebsiella pneumoniae*, and *Candida* spp., have been identified as the pathogens that are most often responsible for healthcare-associated infections (HAIs) [7].

These pathogens produce extracellular polymer material, which constitutes the initial phase of the microbial colonization cycle. While attachment can be reversible at this stage due to physical surface–microbe interactions, it becomes irreversible once the bacterial proteins are segregated [8]. This is followed by microcolony formation, biofilm maturation, and, ultimately, film dispersion [9,10], leading to colony proliferation and host infestation. Specific polymers have been modified to act as antibiofouling agents to interrupt this cycle. Some of the most used polymers in biomaterials in the last decade are poly(N-isopropyl acrylamide), or poly(NiPAAm), and poly(ethylene glycol), or PEG [11]. Poly(NiPAAm) presents the capacity for thermosensitive transitions. For this reason, it has been used in controlled drug release systems. On the other hand, a disadvantage is its toxicity in biological components [12]. This effect is due to the high chemical reactivity of chemical structures with amide groups and their ability to form hydrogen bonds with proteins and nucleic acids in biological systems [13]; consequently, the long-term use of polymers or chemical structures focused on biomedical applications can cause structural and functional alterations at the cellular level [14]. Thus, the development of new systems for drug release that address their toxicity must be evaluated with in vitro or in vivo biological models [15]. In contrast, PEG has the characteristic of increasing the stability of the material when in contact with a biological system. Its strong capacity to reduce an organism’s immunological response or opsonization makes it especially important to prolong its half-life in systemic circulation, and it is excellent for biomedical applications [16]. Another essential characteristic of PEG is its strong capacity to act as an antifouling agent. PEG’s high hydrophilicity results in robust hydration and steric hindrance effects. When applied as a coating to a surface, PEG can create a hydrophilic layer that hinders the adherence of organisms [17]. Therefore, PEG is an essential component of medical devices, particularly due to its high strength and durability. These devices are constantly exposed to mechanical loading during their use. For example, orthopedic implants, suture threads, catheters, and dialysis probes are subjected to constant mechanical forces in the body or at the application site. The use of polymers with good mechanical properties ensures that these devices are strong enough to withstand the stresses and loads to which they are exposed, contributing to their long-term durability [18,19]. In previous works, systems have been developed from PEG and PNiPAAm [20], focusing mainly on their synthesis, characterization, and some mechanical testing. Previous synthesis research has focused on chemical initiation via various routes, such as atom transfer radical polymerization (ATRP) and reversible deactivation radical polymerization (RAFT) [21]. However, in the literature, there is a limited focus on antimicrobial activity and its effect on biological systems. On the other hand, the biological evaluation of systems obtained from ionizing radiation (gamma rays) has not been reported. Gamma irradiation is a strategy adopted to control the degree of crosslinking of polymer chains. Depending on the dose and duration of radiation, the degree of crosslinking can be modulated, and the mechanical properties of the polymers, such as their tear resistance, tensile strength, and hardness, can be improved [22]. In this work, the mechanical and biological properties of IPNs based on a copolymer obtained in two stages (the first being gamma radiation) and consisting of poly(ethylene glycol)/poly(n-isopropylacrylamide) are evaluated in terms of their potential for biomedical applications. These copolymers have previously been used for drug delivery systems in hydrogels and scaffolds (tissue engineering). Their mechanical properties and toxicity are common problems in this context [20,23].

## 2. Materials and Methods

### 2.1. Materials

Poly(ethylene glycol) (PEG) at 6000 and 20,000 Mn was purchased from Wako Pure Chemical Industries, LTD. (Osaka, Japan), and it was used as received. The monomer N-isopropylacrylamide (NiPAAm) and ammonium persulfate (APS), N,N,N’,N’-tetramethylethylendiamine (TEMED), and tetraethylene glycol dimethacrylate (4EGDMA) were acquired from Sigma Aldrich (Naucalpan de Juarez, Mexico). TEMED and 4EGDMA were purified by passing them through an aluminum column. NiPAAm was recrystallized from a 7:3 hexane/toluene solution mix, and this process was repeated twice. Then, it was dried under a vacuum for 24 h. The synthesis of net-PEG and net-PEG-inter-net-NiPAAm (IPN systems) has been previously reported [24]. A brief explanation of the procedure can be found in the following paragraphs.

### 2.2. Synthesis

As the first step, argon gas was bubbled in an aqueous solution of PEG (20 kDa, 50 g/dL) placed in Pyrex ampoules for 20 min. After oxygen displacement, the ampoules were sealed. They were placed in a dewar with an ice bath (−4 °C) to be irradiated with a 60 Co gamma-ray source (Gammabeam 651-PT, Nordion International Inc., Ottawa, Canada) at a dose rate of 9.3 kGy/h. The crosslinked gel (80%) was extracted in water for at least 24 h. Then, net-PEG was dried under a vacuum at a constant temperature and stored. The next step was IPN synthesis through chemical reaction. net-PEG was swollen in a NiPAAm aqueous solution (1, 0.6, and 0.3 M) for 24 h; afterwards, the system was placed under an Ar atmosphere, bubbling the gas for 15 min, and then it was placed in contact with 4EGDMA, TEMED, and APS. The reaction occurred at 70 °C for 1 and 5 h with the desired interpenetration percentage. The PNiPAAm homopolymer and the reaction residues were extracted with water (48 h) to eliminate the non-crosslinked NiPAAm.

### 2.3. Characterization

#### ^13^C Solid-State Nuclear Magnetic Resonance 

NMR testing was performed through the ^13^C in the solid state, using the total lateral band suppression (CP/TOSS) technique. Hydrogel networks were packed in zirconia rotors with a 4 mm diameter. The spinning rate was 5 kHz. 

### 2.4. Mechanical Tests

The net-PEG and IPNs were swollen in deionized water for the maximum swelling time. To assess the purely elastic behavior of the hydrogels, the Young’s modulus (E) and long-term stiffness (E_∞_) of the relaxation modulus (E(t)) were used. Relaxation and indentation tests were conducted using the FT-MTA03 Micromechanical Testing and Assembly System (FemtoTools AG, Zürich, Switzerland), as described in [25]. Data were obtained with an FT-S200 tip (spherical tip with a 50 µm diameter) with a measurement range of ±200 µN and a maximum resolution of 0.0005 µN (at a sampling rate of 10 Hz).

Force vs. displacement curves were obtained with indentation velocities ranging from 1 to 50 µm/s, with a sampling frequency of 100 Hz and a 0.2 µm indentation step. As soft gels were measured, a force limit of 171 µN was set for the experiments. Time vs. force data were collected when the tip was programmed to halt its vertical movement and remain static at a fixed indentation depth for the duration of the force measurement. The relaxation tests involved an indentation phase at a predetermined speed of approximately 100 µm/s, stopping when the force sensor detected a user-defined force value between 2 and 50 µN. The force was then sampled at a rate of 100 Hz for a period of 15 to 60 s.

The data analysis was performed using custom-made programs in Python 3.x (Google Colab, Mountain View, CA, USA), designed to compute the elastic modulus (microindentation tests) as well as the more complex constitutive mechanical properties (relaxation tests) of the materials under test. The relaxation data were fitted to the generalized Maxwell model as described in [25].

### 2.5. Biological Tests

#### 2.5.1. Antimicrobial Test 

The antimicrobial efficacy assessment involved examining Gram-positive bacteria (*Staphylococcus aureus* and *Staphylococcus epidermidis*) and Gram-negative (*Escherichia coli*) bacterial strains. First, 20 mg of the IPN was carefully deposited into tubes containing Mueller–Hinton agar solution. The bacterial concentration was adjusted to 1.5 × 108 CFU/mL (colony-forming units per milliliter) of three bacteria (each independent), adhering to the McFarland standard. Subsequently, the prepared samples underwent an incubation period at 37 °C for 24 h. After the incubation period, the samples were subjected to UV–Vis spectrophotometry, measuring the absorbance at a wavelength of 600 nm. It is important to note that all experiments were meticulously conducted in triplicate to ensure the results’ reliability and consistency.

#### 2.5.2. Antibiofouling Tests (Ovalbumin Adsorption)

Samples of interest (20 mg) were immersed in a 5 mL ovalbumin solution (22 mg/mL). After specified periods, the absorbance values were read (*λ* = 280 nm) with a sample of the supernatant solution. The absorbed protein was calculated due to the change in the concentration in the solution, considering that if this value was lower than the initial one, the ovalbumin was adsorbed on the hydrogel surface.

#### 2.5.3. Cytotoxicity Test 

**Figure 1:**
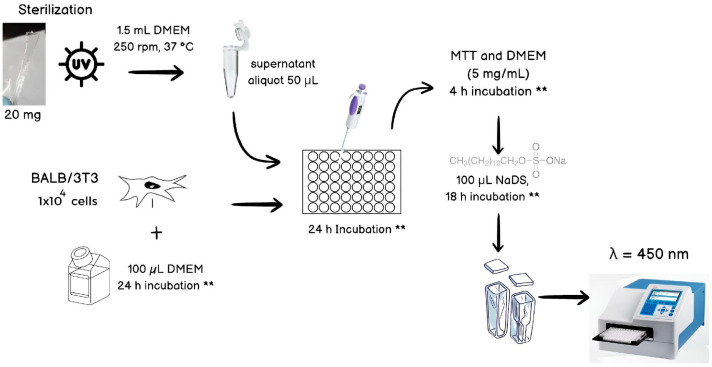
Illustration of the cytotoxicity assay scheme, ** Incubation conditions: 37 °C, 5% CO_2_.

A sample of interest (20 mg) was exposed to UV light for sterilization. Afterwards, the sample was submerged in an Eppendorf tube containing 1.5 mL of Dulbecco’s Modified Eagle’s Medium (DMEM). The tubes were placed under oscillation (250 rpm, 37 °C, 96 h). BALB/3T3 cells (1 × 104 cell/well) were seeded in a 96-well plate with 100 μL DMEM and were incubated at 37 °C and 5% CO_2_ for 24 h. Following this, 50 μL was transferred from the Eppendorf tube to the well plate. A second incubation period was carried out (37 °C, 5% CO_2_), and, after 24 h, an MTT (3-(4,5-dimethylthiazol-2-yl)-2,5-diphenyltetrazolium bromide) and DMEM mixture (5 mg/mL according to the producer) was added. Four hours later, after incubation, 100 μL dissolver buffer solution was incorporated. The incubation conditions were replicated for 18 h, and then a sample was transferred to a vial and assessed in the UV–Vis spectrometer at 450 nm (Figure 1). A comparison of the absorbance values of the samples and those of the cells without hydrogels was performed in order to evaluate the results. The experiments were performed in triplicate. A statistical analysis of the data was performed using a one-way ANOVA with GraphPad Prism 7 (San Diego, CA, USA).

## 3. Results

### 3.1. Characterization

#### ^13^C Solid-State Nuclear Magnetic Resonance (CP/TOSS NMR)

**Figure 2:**
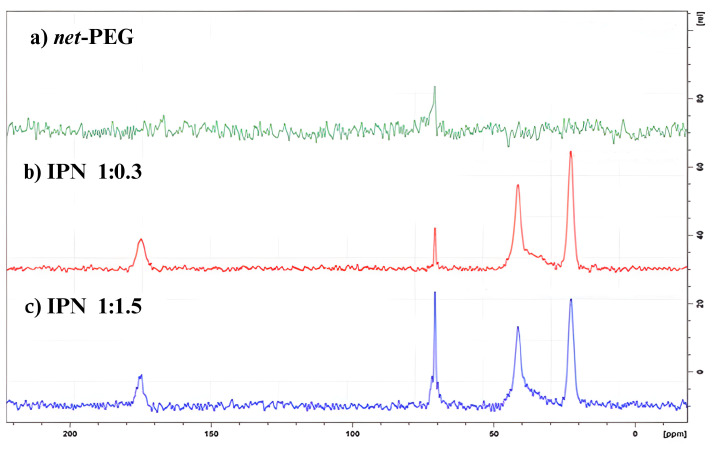
NMR spectra in the solid state of (**a**) the PEG hydrogel without interpenetration, i.e., net-PEG; (**b**,**c**) IPNs with different PNiPAAm concentrations.

As a complementary technique to strengthen the characterization of the IPNs, a ^13^C solid-state NMR (CP/TOSS) analysis was performed. Figure 2 shows the results of the chemical characterization using NMR for net-PEG and two IPNs. In the first spectrum, the peak at 70 ppm corresponds to a methylene -CH-. In the spectra of the IPNs, it is possible to observe three new peaks from NiPAAm: the first one, at 175 ppm, matches the displacement expected for the carbonyl carbons; the second one is found at 40 ppm and arises from the methylene carbon after the polymerization of the vinyl group in the monomer; finally, the peak at 20 ppm corresponds to the methyl from the isopropyl group in the structure [26]. This result confirms the polymerization process of NiPAAm in the IPN. This was difficult to verify in previous FTIR experiments. Due to the high intensity of the amide carbonyl (C=O) stretching and N-H bending bands, they overlap with the C=C stretching region.

### 3.2. Mechanical Tests

As mentioned previously, the mechanical properties of surfaces play a significant role in the attachment of microorganisms. These mechanical characteristics are also intricately connected to the pore size and crosslinking degree. The measurement of the pore size (as depicted in the supplementary materials of our previous study) revealed that a loss of regularity occurs when there is a more significant degree of PNiPAAm interpenetration [24]. Consequently, considering that the microindentation technique was performed in a localized manner and at the microscale, the mechanical properties varied accordingly. The results showed that the stiffness increased as the amount of PNiPAAm in the IPN increased.

**Figure 3:**
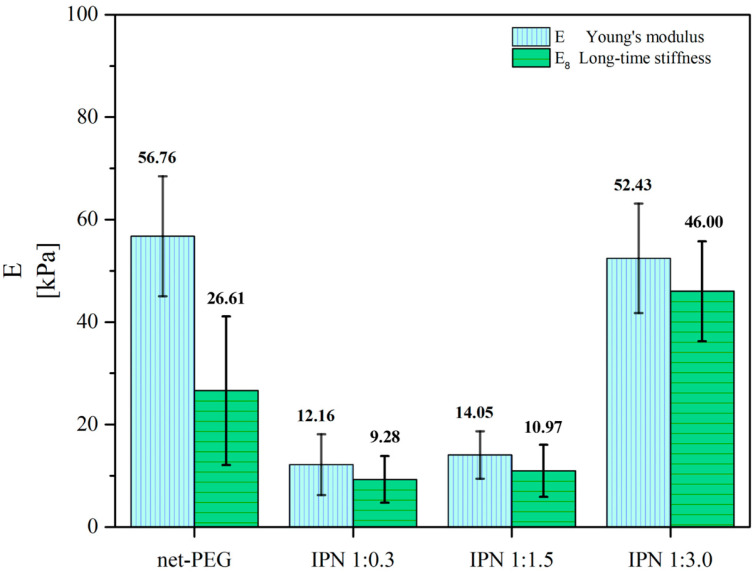
Comparison of the hydrogels’ purely elastic behavior, the Young’s modulus, and the storage modulus.

Table 1 presents the Young’s modulus value (E), which corresponds to the “ideal elastic” behavior of the hydrogels. However, as hydrogels have viscoelastic characteristics, their performance should be considered over time. Therefore, the long-term stiffness (E_∞_) is also reported as it represents the purely elastic behavior of the viscoelastic material. This information is also visualized in Figure 3. This figure shows an increase in the elastic modulus with the amount of interpenetration, ranging from 12.16 kPa (1:0.3 ratio) to 52.43 kPa (1:3 ratio). This indicates the IPN’s greater stiffness and its enhanced capacity to withstand elastic loads. Additionally, the increase in the long-term stiffness modulus, from 9.28 kPa to 46.00 kPa, suggests the IPN’s greater ability to resist sustained deformation and maintain its structural integrity over time. These promising results suggest that the IPN could have important applications in medical/surgical procedures where high stability is required, considering how it is handled by medical personnel and the swelling characteristics that are dependent on the application area. 

### 3.3. Biological Tests

#### 3.3.1. Antimicrobial Test

**Figure 4:**
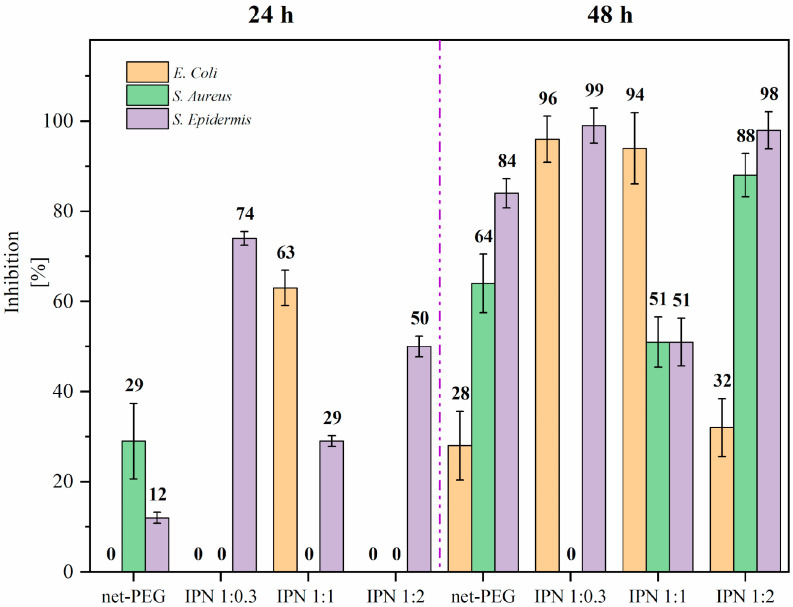
The responses of bacteria in contact with net-PEG and IPNs, measured 24 and 48 h later.

The activity of three bacterial strains was investigated after their interaction with the synthesized IPN systems. If our material behaved as expected, the IPN surfaces could avoid colonization by the bacteria. The results are shown in Figure 4. It was observed that at 24 h, neither the single net-PEG network nor the IPNs demonstrated the total inhibition of the bacteria considered in this study. This is attributed to the low swelling of the gel after 24 h, its degree of crosslinking, and the specific lower critical solution temperature (LCST) ([24]). In contrast, after a 48 h exposure period, all hydrogels showed activity against Gram-positive and -negative bacteria. PEG’s inhibition is highly dependent on its molecular weight. Longer chains can adopt more extended and flexible conformations, which is beneficial in maximizing the surface coverage and reducing bacterial adhesion [27]. It can be seen that the inhibition increases after 48 h because, in some cases, prolonged exposure generates a stable layer (an antifouling layer). The IPN’s inhibition at 48 h depends on factors such as the degree of swelling during copolymerization with poly(n-isopropylacrylamide), which was demonstrated to have an LCST between 31 and 34.5 °C [21]. Likewise, due to the synergy with the antifouling effect of PEG in most experiments, the inhibition of *Staphylococcus epidermidis* is very promising, as it is a common bacterium that forms biofilms on medical devices such as prostheses and catheters, as well as being associated with nosocomial infections [28]. The IPN with a ratio of 1:0.3 showed outstanding results regarding a significant number of positive (*S*. *epidermidis*) and negative (*E. coli*) bacteria. PEG, as an unmodified polymer, did not show an inhibitory effect on any of the tested microorganisms.

#### 3.3.2. Antibiofouling Tests (Ovalbumin Adsorption)

**Figure 5:**
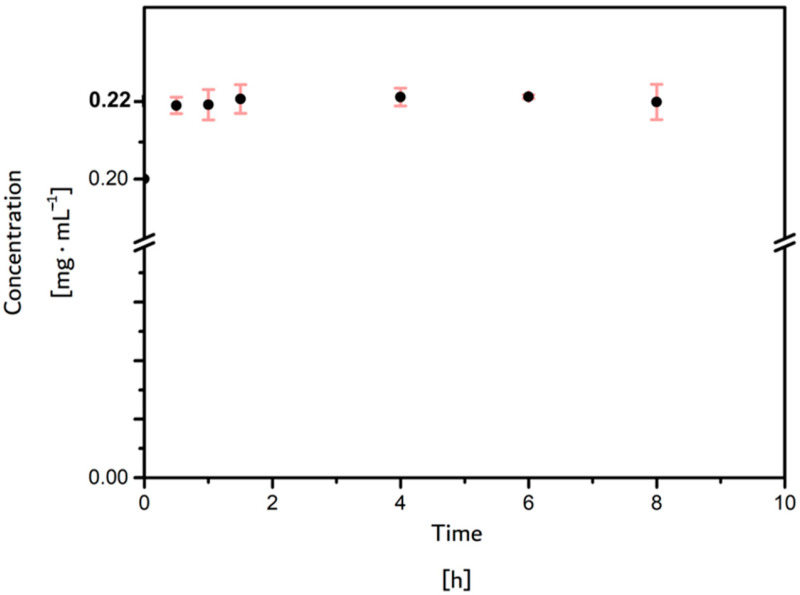
Stable ovalbumin concentration throughout the time period tested when in contact with IPN 1:0.3.

The ovalbumin adsorption test is often used to evaluate the ability of a material to resist protein adsorption, which is a critical stage in the biofouling process [29]. The ovalbumin concentration was stable throughout the period tested when in contact with the IPN at 1:0.3. Ovalbumin was employed as a model protein in contact with the IPN at 1:0.3. The protein concentration was monitored through UV–vis spectrophotometry. Figure 5 illustrates the results, indicating the absence of protein attachment to the IPN’s surface (Figure 5).

#### 3.3.3. Cytotoxicity Test

**Figure 6:**
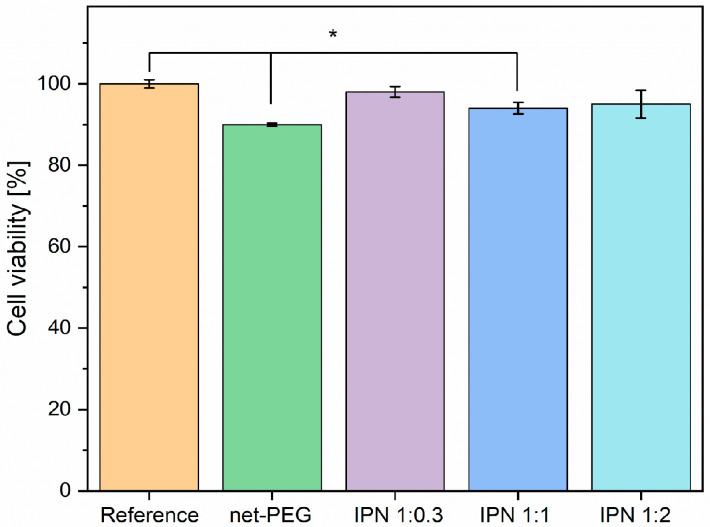
Cell viability of BALB/3T3 cells when seeded onto net-PEG and IPNs. Asterisks denote statistical significance between samples (*p* < 0.05).

One of the main objectives of this study was to develop a material that is resistant to pathogen adhesion and exhibits biocompatibility. Cell culture was the starting point in obtaining toxicological information. In the first stage, the initial materials and the IPN were exposed to BALB cells for 24 h to evaluate their viability and proliferation. In Figure 6, we present the compounds evaluated in groups and in triplicate for net-PEG and the various IPNs. The results showed that the materials were cytocompatible as none of them showed less than 90% cell viability when compared with the control (cells exposed to the culture medium). It was found that the IPNs were similar between the groups studied and the control; conversely, the net-PEG material showed a statistically significant difference (Figure 6). However, they also exhibited the percentage necessary to be considered biocompatible according to the international [30]. The fulfillment of this criterion is necessary for medical devices and their biomedical applications, such as in wound dressings, cellular scaffolds, or catheter coatings. Therefore, the materials were considered suitable for further testing.

## 4. Conclusions

The degree of mechanical resistance of the IPNs could be controlled depending on the degree of copolymerization of PEG and PNiPAAm. This aspect is highly important depending on the type of medical device, the implantation site in the patient, and the rigidity–stress needs in the face of constant manipulation. The IPN with a ratio of 1:03 presented interesting results in terms of bacterial inhibition, as it exhibited strong inhibitory effectiveness in Gram-positive and Gram-negative strains. The antifouling effect of the IPNs was observed in the bacterial inhibition tests after 48 h. Moreover, the ovalbumin adsorption model could be corroborated. These IPNs are good candidates for the development of biocompatible materials as their cell viability values when using BALB/3T3 cells (murine fibroblasts) were above 80%, essentially classifying the materials as cytocompatible. The IPNs obtained are sensitive and biocompatible (in vitro) and represent thermal stimulus systems with promising bacterial inhibition and antifouling effects (of high interest for antimicrobial resistance). Therefore, they can serve as coatings for medical devices in biomedical applications such as medical/surgical procedures and in the design of scaffolds for tissue regeneration.

## Figures and Tables

**Table 1:** Mechanical values obtained for the systems through microindentation.

Sample	PEG:NiPAAm Relation	E [kPa]	E_∞_ [kPa]
net-PEG	1:0	56.76 ± 11.77	26.61 ± 14.57
(net-PEG)-inter-(net-PNiPAAm)	1:0.3	12.16 ± 5.95	9.28 ± 4.55
	1:1.5	14.05 ± 4.64	10.97 ± 5.09
	1:3	52.43 ± 10.72	46.00 ± 9.75

## Data Availability

The original contributions presented in the study are included in the article, further inquiries can be directed to the corresponding author.
